# Response and participation of underserved populations after a three-step invitation strategy for a cardiometabolic health check

**DOI:** 10.1186/s12889-015-2139-x

**Published:** 2015-09-03

**Authors:** Iris Groenenberg, Mathilde R. Crone, Sandra van Dijk, Jamila Ben Meftah, Barend J. C. Middelkoop, Willem J. J. Assendelft, Anne M. Stiggelbout

**Affiliations:** Department of Public Health and Primary Care, Leiden University Medical Center, Hippocratespad 21, PO Box 9600, V0-P, 2300 RC Leiden, The Netherlands; Department of Primary and Community Care, Radboud University Medical Center, Nijmegen, The Netherlands; Department of Medical Decision Making, Leiden University Medical Center, 2300 RC Leiden, The Netherlands

## Abstract

**Background:**

Ethnic minority and native Dutch groups with a low socioeconomic status (SES) are underrepresented in cardiometabolic health checks, despite being at higher risk. We investigated response and participation rates using three consecutive inexpensive-to-costly culturally adapted invitation steps for a health risk assessment (HRA) and further testing of high-risk individuals during prevention consultations (PC).

**Methods:**

A total of 1690 non-Western immigrants and native Dutch with a low SES (35–70 years) from six GP practices were eligible for participation. We used a ‘funnelled’ invitation design comprising three increasingly cost-intensive steps: (1) all patients received a postal invitation; (2) postal non-responders were approached by telephone; (3) final non-responders were approached face-to-face by their GP. The effect of ethnicity, ethnic mix of GP practice, and patient characteristics (gender, age, SES) on response and participation were assessed by means of logistic regression analyses.

**Results:**

Overall response was 70 % (*n* = 1152), of whom 62 % (*n* = 712) participated in the HRA. This was primarily accomplished through the postal and telephone invitations. Participants from GP practices in the most deprived neighbourhoods had the lowest response and HRA participation rates. Of the HRA participants, 29 % (*n* = 207) were considered high-risk, of whom 59 % (*n* = 123) participated in the PC. PC participation was lowest among native Dutch with a low SES.

**Conclusions:**

Underserved populations can be reached by a low-cost culturally adapted postal approach with a reminder and follow-up telephone calls. The added value of the more expensive face-to-face invitation was negligible. PC participation rates were acceptable. Efforts should be particularly targeted at practices in the most deprived areas.

**Electronic supplementary material:**

The online version of this article (doi:10.1186/s12889-015-2139-x) contains supplementary material, which is available to authorized users.

## Background

Cardiometabolic disease (CMD), namely cardiovascular disease (CVD), diabetes mellitus (DM), and kidney failure, is a leading cause of death in high-income countries [1]. CMD risk is related to low socioeconomic status (SES) and a non-Western origin [2, 3]. In The Netherlands, CVD prevalence and mortality are particularly high among Surinamese and Turkish people [4, 5]. Turkish, Moroccans, and especially Hindustani Surinamese have a higher DM risk [6]. As CMD is largely preventable, focus has shifted towards primary prevention among high-risk individuals and, as a result, health checks have been implemented in various countries [7–9]. A non-Western origin and a low SES are associated with lower health check attendance [10]. This selective non-attendance contributes to inequalities in health gains from screening. Efforts to increase participation of these underserved (difficult-to-reach, high-risk) populations are therefore relevant, and a prerequisite for cost-effectiveness [11, 12].

Attempts to increase participation in health checks in the general population usually compared postal, telephone, and face-to-face strategies in parallel [13–17]. In general, a postal invitation combined with telephone reminders was most effective in cancer screening attendance [14]. However, studies taking ethnicity or SES into account tend to find the more labour-intensive, expensive face-to-face strategies or combinations of strategies, to be most effective [13, 15–17]. In The Netherlands, only this ‘case-finding’ approach is currently reimbursed by basic health insurance [18]. Nevertheless, a strategy with a sequential inexpensive-to-costly ‘funnel’ invitation procedure might be more cost-effective. We investigated response and participation in a health check by using such a funnel design that encompassed three consecutive culturally targeted and personalised invitation steps: first, a postal invitation to eligible individuals, second, a telephone invitation for postal non-responders, third, a face-to-face invitation for telephone non-responders. We assessed both response and participation, with response referring to the patient’s awareness of the screening and providing a response as to whether or not (s)he intended to participate, and participation to actual participation in the health check.

Another way of increasing cost-effectiveness entails using a two-stage health check approach, which usually refers to employing a non-invasive and low-cost risk stratification tool for all individuals, followed by more expensive biometric and blood testing for high-risk individuals [12, 19]. The Dutch cardiometabolic health check follows such a two-stage approach. Stage one comprises a short health risk assessment (HRA) consisting of six risk factor questions [20, 21] for people aged 45–70 years. Patients have to calculate their own HRA risk score. In case of an increased risk according to the HRA, patients are advised to attend a prevention consultation (PC) at the GP (stage two). However, in the general population it has been shown that patients then refrain from participation on two separate occasions (HRA and PC), possibly leading to even higher non-participation rates among underserved populations [22]. Therefore, we examined HRA participation and subsequent PC participation after receiving an increased HRA risk score, as well as the effect of ethnicity, ethnic mix of GP practice, and patient characteristics (gender, age, SES) on participation.

Summarizing, our research questions were:

1. What are response and participation rates among different underserved populations after a postal invitation to complete the HRA?
2. To what extent can response and HRA participation among postal non-responders from the different groups be increased by telephone and by a subsequent face-to-face invitation by the GP among remaining non-responders?
3. What proportion of high-risk HRA participants attends the PC, and does this vary between different underserved populations and invitation steps?

## Methods

### Study population and setting

Between May 2012 and December 2013, patients from six general practices in deprived neighbourhoods in the Netherlands were invited for the cardiometabolic health check. Patients had to be Turkish, Moroccan, or Surinamese, or native Dutch with a low SES. As ethnicity is not registered by the GP, ethnic origin was deduced from family name, after which the classification was checked by the GP. He/she also selected the native Dutch patients with a low SES. The SES status was then corroborated by a neighbourhood SES score. A low SES score represents a low neighbourhood social status and consists of the average income and the proportion of low-income, low-educated, and unemployed individuals [23]. Patients had to be 45–70 years old. The lower age limit for the Hindustani Surinamese was 35 years because of their genetically increased risk of DM. Exclusion criteria were having (had) CMD, use of antihypertensive/lipid-lowering drugs, or having a complete cardiometabolic risk profile within the previous year (see Additional file 1).

Ethical approval was obtained from the Medical Ethics Committee of the Leiden University Medical Centre. Participation in the study followed an ‘opt-out procedure’: patients could sign a reply card declining participation.

### Three-step invitation strategy for stage one: HRA participation

The HRA consisted of six short questions on age, smoking status, BMI, waist circumference, and family history of CVD or DM. Three culturally targeted and personalised invitation steps for the HRA were tested following a funnel design.

- Step one: eligible patients were invited by a personalised, GP-signed letter. Enclosed were the HRA and an information brochure (both with ethnic specific pictures), a tape measure for measuring waist circumference, a reply card declining participation, and a stamped return envelope addressed to the GP. The formulation was simplified to fit the generally lower health literacy levels of our target population. Turkish and Moroccan patients received Turkish or Arabic versions, respectively, in addition to the Dutch materials. After two weeks of non-response, patients received a reminder package. A detailed description of the (cultural) adaptations made in the invitation, HRA, and information brochure can be found in Additional file 2.
- Step two: after another two weeks of non-response, patients were called by a trained research assistant on behalf of the GP. Turkish and Moroccan patients were called by Turkish, Arabic, and Berber (which is an oral only language) speaking research assistants. The conversation was structured by a script supporting patients in making an informed decision about (non-)participation. When a participant decided to participate, the HRA was immediately completed by telephone and the HRA risk score was calculated by the research assistant. The national telephone directory was consulted when telephone numbers were missing, unlisted, or inoperative. Patients were approached with a maximum of four call attempts.
- Step three: after four failed call attempts, patients were invited face-to-face when visiting their GP for an unrelated consultation. GPs received a pop-up in the electronic patient file of a non-responding patient. The GPs followed a short version of the telephone script to help patients make an informed decision about (non-)participation. When a participant decided to participate, the HRA was immediately completed at the GP practice and the HRA risk score was calculated by the practice nurse. The face-to-face invitation period lasted six months, which was deemed long enough since ethnic minorities and native Dutch patients with a low SES are known to consult the GP up to once or twice a month [24, 25]. If patients had not visited the practice within this period, they were classified as final non-responders.

### Stage two: PC participation among high-risk individuals

Participants had to calculate their own HRA risk score. Participants with a low risk score were referred to the Dutch health check website where advice for maintaining or improving their lifestyle was provided. Participants with a high-risk score were advised to attend the PC. This advice was provided either written, by phone, or face-to-face, depending on the relevant invitation step. Patients themselves were responsible for making an appointment with the GP. During the first PC, the biometric HRA measures were checked (weight, height, and blood pressure) and lab work on fasting glucose and cholesterol levels was completed. During the second PC, the results were discussed, the cardiometabolic risk profile was drawn, lifestyle advice was provided, and medication was prescribed if necessary [26]. Because we only looked at participation in the first consultation, we refer to both consultations as one (‘PC participation’).

### Measures

The main outcome measures were response, HRA participation, and PC participation. The secondary outcome measure was HRA risk score.

*Response* was defined as ‘yes’ if an individual provided a reaction as to whether he/she wanted to participate in the HRA or not and ‘no’ if an individual did not respond at all. It was calculated as a percentage of all patients. Telephone response was calculated as the proportion of postal non-responders, who picked up the phone and indicated an intention to participate or not. Finally, face-to-face response was calculated as the proportion of telephone non-responders, who were approached face-to-face by their GP and indicated an intention to participate or not. Additionally, to take into account the fact that not all patients visited their GP for an unrelated consultation in the research period, face-to-face response was also calculated as a percentage of those telephone non-responders who actually visited their GP.

*HRA participation* was defined as ‘yes’ if the HRA was completed and ‘no’ if the HRA was not completed. It was calculated as the proportion of responders of each specific invitation step.

*HRA risk score* was defined as low or high risk and was calculated as the proportion of HRA participants.

*PC participation* was defined as ‘yes’ if the PC was attended when having a high-risk HRA score and ‘no’ if the PC was not attended. It was calculated as the proportion of individuals with a high-risk HRA score.

### Covariates

Patient characteristics were: ethnicity (native Dutch/Turkish/Moroccan/Surinamese), gender (male/female), age (30-45/45-50/50-55/55-60/60-65/65+ years), and neighbourhood SES score (>0/0 till −2/-2 till −4/<−4). A low SES score equals a low SES. The average SES score in the Netherlands in 2010 was 0.17 (−7.25 till 3.19), whereas in our study it was −2.14 (−6.23 till 2.88) [23]. The ethnic mix of GP practice variable was divided in three groups: predominantly non-Western patient population, approximately equal combination, and predominantly native Dutch with a low SES patient population. Invitation steps were: mail, phone, and face-to-face.

### Data analysis

Descriptive analyses were applied to describe the patient population. Differences in patient characteristics between the ethnic groups were assessed by means of ANOVA. Univariate logistic regression was used to assess whether patient characteristics were or ethnic mix of GP practice was related to response and participation rates. Odds ratios (ORs) regarding the influence of ethnicity on outcome measures were corrected for relevant covariates (*p*-value <0.05) by means of multivariate logistic regression. As the populations who responded to the various invitation steps logically differed, results were stratified by invitation step.

## Results

### Demographics

Of the 1690 individuals eligible for invitation, 43 had an unknown or wrongly classified ethnicity, two had started antihypertensives right before start of the study, and one had missing contact details. Exclusion from analyses resulted in 1644 eligible individuals. Slightly more males (54 %) than females (46 %) were invited (Table 1). The Moroccan group consisted of more males than the native Dutch and Surinamese groups. Participants were on average 50 years old. The native Dutch were older and the Surinamese were younger than the other ethnic groups. The native Dutch and the Turkish had a higher and a lower SES score than the other ethnic groups, respectively.

**Table 1:** Sociodemographic characteristics of all patients eligible for the cardiometabolic health check

	Total (*n* = 1644)			Dutch with low SES (*n* = 437)			Turkish (*n* = 353)			Moroccan (*n* = 344)			Surinamese (*n* = 510)			
	n	%	mean (SD)	n	%	mean (SD)	n	%	mean (SD)	n	%	mean (SD)	n	%	mean (SD)	*p* value
Gender																
Male	882	54		220	50		192	54		210	61^a^		260	51		.011
Female	762	46		217	50		161	46		134	39		250	49		
Age (years)	1644	100	50 (7.00)	437	100	52 (6.27)^b^	353	100	51 (5.55)	344	100	51 (6.32)	510	100	47 (7.57)^c^	<.001
Age (cat.)																
35-45	259	16		14	3		8	2		15	4		222	44		
45-50	595	36		160	37		164	46		147	43		124	24		
50-55	392	24		118	27		97	27		93	27		84	16		
55-60	213	13		75	17		48	14		44	13		46	9		
60-65	120	7		45	10		26	7		27	8		22	4		
65+	65	4		25	6		10	3		18	5		12	2		
SES score (score)	1644	100	−2.14 (2.46)	437	100	−0.39 (1.55)	353	100	−3.32 (2.28)	344	100	−2.30 (2.43)	510	100	−2.73 (2.43)	<.001^d^
SES score (cat.)																
> 0	470	29		231	53		48	14		82	24		109	21		
0 till −2	386	24		151	35		57	16		91	26		87	17		
−2 till −4	267	16		34	8		66	19		52	15		115	23		
< −4	521	32		21	5		182	52		119	35		199	39		

^a^Significantly more males than females when compared to the native Dutch and Surinamese

^b^Significantly older than the other ethnic groups

^c^Significantly younger than the other ethnic groups

^d^All ethnic groups differed significantly from each other

### Response

**Fig. 1:**
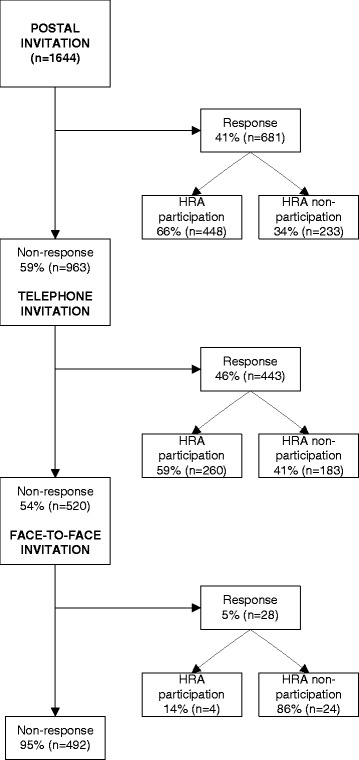
Flowchart response and participation by postal, telephone, and face-to-face invitation step, with response referring to the patient’s awareness of the screening and providing a response as to whether or not (s)he intended to participate, and participation to actual participation in the health check

Total response (those who indicated an intention to participate or not) was 70 % (*n* = 1152) of our underserved populations (Fig. 1). Of all individuals invited, 41 % (*n* = 681) responded to the postal invitation (Table 2). Of the postal non-responders, 46 % (*n* = 443) responded by telephone. Finally, of all telephone non-responders, 5 % (*n* = 28) responded face-to-face. When we only considered those non-responders who attended their GP for an unrelated consultation during the research period of 6 months (*n* = 225), response was 12 %. Face-to-face results are not presented in the tables as numbers were too small. A comparison between (postal or telephone) responders (*n* = 1125) and non-responders (*n* = 520) revealed that those left over for face-to-face recruitment were more often men (*p* ≤ 0.001) and individuals with a low SES score (*p* ≤ 0.001).

**Table 2:** Response in postal and telephone steps

		Postal		Telehone^a^	
		Response	Odds ratio (95 % CI)	Response	Odds ratio (95 % CI)
Total group (*n* = 1644)		41 % (*n* = 681)		46 % (*n* = 443)	
Univariate analyses					
Ethnicity	Dutch^c^ (*n* = 437)	49 % (*n* = 214)	1.00	57 % (*n* = 126)	1.00
	Turkish (*n* = 353)	45 % (*n* = 158)	0.84 (0.64-1.12)	47 % (*n* = 91)	0.67 (0.46-0.99)^*^
	Moroccan (*n* = 344)	39 % (*n* = 134)	0.67 (0.50-0.89)^*^	40 % (*n* = 84)	0.51 (0.35-0.75)^**^
	Surinamese (*n* = 510)	34 % (*n* = 175)	0.54 (0.42-0.71)^**^	42 % (*n* = 142)	0.57 (0.40-0.80)^**^
Gender	Male^c^ (*n* = 882)	39 % (*n* = 343)	1.00	42 % (*n* = 225)	1.00
	Female (*n* = 762)	44 % (*n* = 338)	1.25 (1.03-1.53)^*^	51 % (*n* = 218)	1.48 (1.14-1.91)^*^
Age	35-45 (*n* = 259)	27 % (*n* = 70)	0.51 (0.37-0.70)^**^	46 % (*n* = 87)	0.90 (0.63-1.28)
	45-50^c^ (*n* = 595)	42 % (*n* = 250)	1.00	49 % (*n* = 168)	1.00
	50-55 (*n* = 392)	45 % (*n* = 176)	1.12 (0.87-1.45)	49 % (*n* = 106)	1.02 (0.72-1.43)
	55-60 (*n* = 213)	46 % (*n* = 98)	1.18 (0.86-1.61)	44 % (*n* = 51)	0.84 (0.55-1.28)
	60-65 (*n* = 120)	48 % (*n* = 58)	1.29 (0.87-1.91)	36 % (*n* = 22)	0.58 (0.33-1.02)
	65+ (*n* = 65)	45 % (*n* = 29)	1.11 (0.66-1.85)	25 % (*n* = 9)	0.35 (0.16-0.77)^*^
GP practice^b^	Dutch^c^ (*n* = 361)	50 % (*n* = 179)	1.00	61 % (*n* = 111)	1.00
	Mix (*n* = 193)	54 % (*n* = 105)	1.21 (0.86-1.72)	56 % (*n* = 49)	0.80 (0.48-1.35)
	Non-Western (*n* = 1090)	36 % (*n* = 397)	0.58 (0.46-0.74)^**^	41 % (*n* = 283)	0.44 (0.32-0.62)^**^
SES score	>0^c^ (*n* = 470)	46 % (*n* = 217)	1.00	54 % (*n* = 137)	1.00
	0 till −2 (*n* = 386)	41 % (*n* = 160)	0.83 (0.63-1.08)	47 % (*n* = 106)	0.75 (0.52-1.07)
	−2 till −4 (*n* = 267)	39 % (*n* = 104)	0.74 (0.55-1.01)	35 % (*n* = 57)	0.46 (0.30-0.68)^**^
	< −4 (*n* = 521)	38 % (*n* = 200)	0.73 (0.56-0.94)^*^	45 % (*n* = 143)	0.68 (0.49-0.95)^*^
Multivariate analyses					
Ethnicity	Dutch^d^ (*n* = 437)	49 % (*n* = 214)	1.00	57 % (*n* = 126)	1.00
	Turkish (*n* = 353)	45 % (*n* = 158)	1.43 (0.98-2.08)	47 % (*n* = 91)	1.11 (0.68-1.82)
	Moroccan (*n* = 344)	39 % (*n* = 134)	0.88 (0.64-1.22)	40 % (*n* = 84)	0.66 (0.43-1.01)
	Surinamese (*n* = 510)	34 % (*n* = 175)	1.23 (0.83-1.81)	42 % (*n* = 142)	0.93 (0.57-1.89)

^a^As percentage of postal non-responders

^b^Predominant composition of patient population

^c^Reference category univariate analyses

^d^Reference category multivariate analyses, corrected for relevant variables (gender, age, ethnic mix of GP practice, and/or SES score)

^*^ *p* < 0.05

^**^ *p* < 0.001

The higher odds of response among native Dutch groups disappeared when adjusting for relevant covariates. This was mainly explained by differences regarding ethnic mix of GP practice (Table 3). The native Dutch in predominantly non-Western practices did not respond more often than the other ethnic groups, and even significantly less than the Turkish (OR 0.52, 95 % CI 0.31-0.88, *p* = 0.014). Additionally, response was higher for all ethnic groups in the mixed and predominantly native Dutch practices when compared to the predominantly non-Western practices (Table 2).

**Table 3:** Response and HRA participation, stratified by GP practice and ethnicity

GP practice	Ethnicity	% (n)	Response, %^a^ (n)	HRA participation, %^b^ (n)
Dutch low SES (*n* = 362)	Dutch	74 % (*n* = 270)	82 % (*n* = 221)	70 % (*n* = 154)
	Turkish	3 % (*n* = 10)	80 % (*n* = 8)	63 % (*n* = 5)
	Moroccan	21 % (*n* = 75)	80 % (*n* = 60)	60 % (*n* = 36)
	Surinamese	2 % (*n* = 7)	86 % (*n* = 6)	50 % (*n* = 3)
	*Total*	*100 % (n = 362)*	*81 % (n = 295)*	*67 % (n = 198)*
Mix (*n* = 194)	Dutch	49 % (*n* = 95)	88 % (*n* = 84)	64 % (*n* = 54)
	Turkish	9 % (*n* = 18)	83 % (*n* = 15)	53 % (*n* = 8)
	Moroccan	39 % (*n* = 76)	74 % (*n* = 56)	66 % (*n* = 37)
	Surinamese	3 % (*n* = 5)	80 % (*n* = 4)	100 % (*n* = 4)
	*Total*	*100 % (n = 194)*	*82 % (n = 159)*	*65 % (n = 103)*
Non-Western (*n* = 1091)	Dutch	6 % (*n* = 73)	58 % (*n* = 42)	57 % (*n* = 24)
	Turkish	30 % (*n* = 325)	72 % (*n* = 235)	58 % (*n* = 137)
	Moroccan	18 % (*n* = 194)	55 % (*n* = 106)	56 % (*n* = 59)
	Surinamese	46 % (*n* = 499)	63 % (*n* = 315)	61 % (*n* = 193)
	*Total*	*100 % (n = 1091)*	*64 % (n = 698)*	*59 % (n = 413)*

^a^As percentage of the entire ethnic group

^b^As percentage of responders

### Stage one: HRA participation

Of the 1152 responders, 62 % (*n* = 712) participated in the HRA (Table 4). Participation rates among postal responders (*n* = 448, 66 %) were comparable to those among telephone responders (*n* = 260, 59 %). The participation rate of face-to-face responders was only 14 % (*n* = 4). Just as with response, the ethnic differences in HRA participation disappeared when adjusting for relevant covariates, in particular ethnic mix of GP practice. In the predominantly native Dutch practices, the native Dutch patients participated more often in the HRA than the non-Western patients (Table 3). However, in the predominantly non-Western and mixed practices, the native Dutch had comparable or lower HRA participation rates than the other ethnic groups (not significant).

**Table 4:** Participation rates of responders to postal and telephone steps

		Postal (response *n* = 681)		Telephone (response *n* = 443)	
		Participation	Odds ratio (95 % CI)	Participation	Odds ratio (95 % CI)
Total group (*n* = 1152)		66 % (*n* = 448)		59 % (*n* = 260)	
*Univariate analyses*					
Ethnicity	Dutch^a^ (*n* = 347)	76 % (*n* = 163)	1.00	55 % (*n* = 69)	1.00
	Turkish (*n* = 258)	58 % (*n* = 91)	0.43 (0.27-0.66)^**^	65 % (*n* = 59)	1.52 (0.87-2.65)
	Moroccan (*n* = 222)	60 % (*n* = 81)	0.48 (0.30-0.76)^*^	56 % (*n* = 47)	1.05 (0.60-1.83)
	Surinamese (*n* = 325)	65 % (*n* = 113)	0.57 (0.37-0.89)^*^	60 % (*n* = 85)	1.23 (0.76-2.00)
Gender	Male^a^ (*n* = 576)	64 % (*n* = 218)	1.00	55 % (*n* = 123)	1.00
	Female (*n* = 576)	68 % (*n* = 230)	1.22 (0.89-1.68)	63 % (*n* = 137)	1.40 (0.96-2.05)
Age	35-45 (*n* = 161)	63 % (*n* = 44)	0.86 (0.49-1.49)	57 % (*n* = 50)	0.75 (0.44-1.28)
	45-50^a^ (*n* = 427)	66 % (*n* = 166)	1.00	64 % (*n* = 108)	1.00
	50-55 (*n* = 287)	66 % (*n* = 117)	1.00 (0.67-1.51)	54 % (*n* = 57)	0.65 (0.39-1.06)
	55-60 (*n* = 156)	67 % (*n* = 66)	1.04 (0.64-1.72)	51 % (*n* = 26)	0.58 (0.31-1.09)
	60-65 (*n* = 83)	59 % (*n* = 34)	0.72 (0.40-1.29)	55 % (*n* = 12)	0.67 (0.27-1.63)
	65+ (*n* = 38)	72 % (*n* = 21)	1.33 (0.57-3.13)	78 % (*n* = 7)	1.94 (0.39-9.66)
GP practice	Dutch^a^ (*n* = 295)	79 % (*n* = 141)	1.00	51 % (*n* = 57)	1.00
	Mix (*n* = 159)	72 % (*n* = 76)	0.71 (0.40-1.23)	55 % (*n* = 27)	1.16 (0.59-2.28)
	Non-Western (*n* = 698)	58 % (*n* = 231)	0.38 (0.25-0.57)^**^	62 % (*n* = 176)	1.56 (1.00-2.43)^*^
SES score	>0^a^ (*n* = 364)	70 % (*n* = 152)	1.00	54 % (*n* = 74)	1.00
	0 till −2 (*n* = 268)	71 % (*n* = 113)	1.03 (0.66-1.61)	56 % (*n* = 59)	1.07 (0.64-1.78)
	−2 till −4 (*n* = 169)	71 % (*n* = 74)	1.06 (0.63-1.76)	63 % (*n* = 36)	1.46 (0.77-2.75)
	< −4 (*n* = 351)	55 % (*n* = 109)	0.51 (0.34-0.77)^**^	64 % (*n* = 91)	1.49 (0.92-2.40)
*Multivariate analyses*					
Ethnicity	Dutch^b^ (*n* = 347)	76 % (*n* = 163)	1.00	55 % (*n* = 69)	1.00
	Turkish (*n* = 258)	58 % (*n* = 91)	0.94 (0.50-1.76)	65 % (*n* = 59)	1.09 (0.53-2.22)
	Moroccan (*n* = 222)	60 % (*n* = 81)	0.71 (0.41-1.23)	56 % (*n* = 47)	0.93 (0.52-1.66)
	Surinamese (*n* = 325)	65 % (*n* = 113)	1.31 (0.69-2.49)	60 % (*n* = 85)	0.88 (0.45-1.71)

^a^Reference category univariate analyses

^b^Reference category multivariate analyses, corrected for relevant variables (ethnic mix of GP practice and/or SES score)

^*^ *p* < 0.05

^**^ *p* < 0.001

### Stage two: HRA risk result and PC participation

Of the HRA participants, 29 % (*n* = 207) had a high-risk result (Table 5). When correcting for relevant covariates, the significantly lower risk score of Surinamese participants disappeared. This was mainly explained by age differences between groups. For Hindustani Surinamese, the age threshold to be invited for the HRA was lower due to their genetic higher risk of DM. The risk formula, however, was not adjusted for this heightened risk.

**Table 5:** HRA risk score and participation in PC

		HRA risk score		Participation in PC^a^	
		High	Odds ratio (95 % CI)	Yes	Odds ratio (95 % CI)
Total group (*n* = 714)		29 % (*n* = 207)		59 % (*n* = 123)	
*Univariate analyses*					
Ethnicity	Dutch^c^ (*n* = 232)	35 % (*n* = 82)	1.00	46 % (*n* = 38)	1.00
	Turkish (*n* = 150)	37 % (*n* = 56)	1.09 (0.71-1.67)	68 % (*n* = 38)	2.44 (1.20-4.97)^*^
	Moroccan (*n* = 132)	30 % (*n* = 40)	0.80 (0.50-1.26)	68 % (*n* = 27)	2.41 (1.09-5.31)^*^
	Surinamese (*n* = 200)	15 % (*n* = 29)	0.31 (0.19-0.50)^**^	69 % (*n* = 20)	2.57 (1.05-6.32)^*^
Gender	Male^c^ (*n* = 344)	38 % (*n* = 130)	1.00	63 % (*n* = 82)	1.00
	Female (*n* = 370)	21 % (*n* = 77)	0.43 (0.31-0.60)^**^	53 % (*n* = 41)	0.67 (0.38-1.18)
Age	35-45 (*n* = 95)	2 % (*n* = 2)	0.14 (0.03-0.61)^*^	0 % (*n* = 0)	-
	45-50^c^ (*n* = 277)	13 % (*n* = 36)	1.00	67 % (*n* = 24)	1.00
	50-55 (*n* = 174)	28 % (*n* = 48)	2.55 (1.57-4.13)^**^	69 % (*n* = 33)	0.91 (0.36-2.29)
	55-60 (*n* = 93)	55 % (*n* = 51)	8.13 (4.75-13.92)^**^	53 % (*n* = 27)	0.51 (0.23-1.16)
	60-65 (*n* = 47)	91 % (*n* = 43)	71.97 (24.37-212.50)^**^	58 % (*n* = 25)	0.63 (0.27-1.49)
	65+ (*n* = 28)	96 % (*n* = 27)	180.75 (23.82-1371.33)^**^	52 % (*n* = 14)	0.49 (0.19-1.29)
GP practice^b^	Dutch^c^ (*n* = 198)	31 % (*n* = 62)	1.00	53 % (*n* = 33)	1.00
	Mix (*n* = 103)	37 % (*n* = 38)	1.28 (0.78-2.12)	50 % (*n* = 19)	0.88 (0.39-1.97)
	Non-Western (*n* = 413)	26 % (*n* = 107)	0.77 (0.53-1.11)	66 % (*n* = 71)	1.73 (0.91-3.29)
SES score	>0^c^ (*n* = 227)	29 % (*n* = 65)	1.00	55 % (*n* = 36)	1.00
	0 till −2 (*n* = 173)	32 % (*n* = 56)	1.19 (0.78-1.83)	55 % (*n* = 31)	1.00 (0.49-2.05)
	−2 till −4 (*n* = 112)	31 % (*n* = 35)	1.13 (0.69-1.85)	60 % (*n* = 21)	1.21 (0.52-2.78)
	< −4 (*n* = 202)	25 % (*n* = 51)	0.84 (0.55-1.29)	69 % (*n* = 35)	1.76 (0.82-3.80)
*Multivariate analyses*					
Ethnicity	Dutch^d^ (*n* = 232)	35 % (*n* = 82)	1.00	NA	NA
	Turkish (*n* = 150)	37 % (*n* = 56)	1.59 (0.93-2.70)	NA	NA
	Moroccan (*n* = 132)	30 % (*n* = 40)	0.92 (0.52-1.63)	NA	NA
	Surinamese (*n* = 200)	15 % (*n* = 29)	0.54 (0.28-1.01)	NA	NA

*NA* Not applicable

^a^As percentage of individuals with a high HRA risk score

^b^Predominant composition of patient population

^c^Reference category univariate analyses

^d^Reference category multivariate analyses, corrected for relevant variables (gender, age)

^*^ *p* < 0.05

^**^ *p* < 0.001

Of the high-risk individuals, 59 % (*n* = 123) participated in the PC. All non-Western groups had higher odds of PC participation when compared to the native Dutch. We found no differences in risk score and in PC participation between the postal versus the telephone step.

## Discussion

### Strengths and weaknesses

We developed materials matching the (cultural) preferences of underserved populations facilitating response and HRA participation possibilities. These adjustments were based on information derived from the literature and the results of focus groups [27]. This approach, combined with the funnelled invitation design, gave as many individuals as possible the opportunity to make an informed decision about participation, acknowledged previously to be important but difficult to measure [28, 29]. With the fast rise of individuals having access to internet we considered using the current online HRA, but after careful deliberation with the populations under study decided it would be fruitless [30]. The pragmatic stepwise invitation approach is most feasible to implement in practice and has the greatest potential of being cost-effective. However, we cannot conclude which invitation step is most effective and, therefore, results are difficult to compare with others usually comparing strategies in parallel. Second, we did not receive a response of 30 % of the patients. In the scope of reducing health inequalities, it is important to reach precisely those individuals about whom we have no health risk information at all, to find out whether our responders are the groups at highest risk. Third, the HRA was completed by participants themselves, possibly leading to reporting errors and mistakes in calculating one’s risk score. Fourth, the telephone calls were performed by research assistants, not the GP practice nurse. The average duration of these calls was nine minutes, however, this included the time necessary to ask some additional questions needed for the study. Approximately six minutes were used to invite a person to participate in the HRA and to complete the HRA. The feasibility of this invitation step in the GP practice needs to be studied further. Finally, the number of GP practices was small because we aimed to recruit practices consisting mainly of specific underserved populations. Therefore, it was impossible to perform multi-level analyses. Theoretically, many practice-level characteristics could influence response and participation, therefore, our conclusions on the effect of practice on outcome measures should be regarded as a first indication and need to be studied further.

### Comparison with other studies

Our postal HRA participation rate was lower compared to the general population [31–33]. This may, in part, be due to the low percentage of underserved populations in other studies and their use of an additional online HRA. Moreover, in these studies HRA results could not be calculated by patients themselves, returning the HRA might have worked as an incentive. In contrast, a pilot study of the Dutch cardiometabolic health check provided the risk score immediately and found similar participation rates as we did [34].

The telephone invitation increased the number of people making a decision about participation. This is in line with a study among non-participants in cardiovascular screening in which 40 % changed their initial decision after receiving additional information about risks and screening [35].

The literature suggests that, if used as a separate strategy, face-to-face strategies are more effective in reaching underserved populations. We found that if used as an additional step in a multi-step strategy, the added value of the face-to-face invitation was negligible. We also saw that the individuals left over for face-to-face recruitment were more often the ‘harder-to-reach’ men and individuals with a low SES. Additionally, face-to-face strategies are labour-intensive and expensive. Given their lack of feasibility in practice and the high response obtained using a postal and telephone invitation, this latter multi-step approach seems advisable [16, 17].

Ethnic differences in response and HRA participation were no longer significant when adjusting for ethnic mix of GP practice, possibly because of differences in practice size or sociocultural aspects (e.g., stronger assimilation and social cohesion in some neighbourhoods). The predominantly non-Western practices had the lowest response and participation rates. These practices were larger and located in more deprived neighbourhoods where social cohesion is usually lower and both native Dutch and non-Western patients may be more illiterate [36]. Unfortunately, we did not have individual SES scores. We did, however, have individual education information for a sample of participants. Using this data did not change our conclusions, justifying the use of a neighbourhood SES score.

The PC participation rate among our high-risk patients was larger than in the pilot study among the general population, but smaller than in two other studies of the Dutch cardiometabolic health check [31, 32, 34]. In the latter studies, high-risk participants were invited for the PC, whereas in both the pilot and our study, high-risk participants were personally responsible for scheduling an appointment. In follow-up interviews, high-risk participants who had not attended the PC frequently indicated that they had not been aware or had not understood they had to schedule their own appointment. Thus, it would be advisable for these groups to shift the responsibility of making an appointment to the GP.

Our PC participation rate was larger than in the British NHS health check [7, 11]. However, their patients were risk-stratified in advance, and only high-risk individuals were invited. We risk-stratified by means of the HRA. High-risk HRA participants were more likely to also participate in the PC.

The lower age threshold for being invited explained the lower HRA risk score among Surinamese. This emphasizes that a lower threshold is only useful when an ethnicity-based risk score is used [37].

The native Dutch with a low SES refrained most often from PC participation. These groups have been shown to rely less on the GP for lifestyle advice [38].

## Conclusions

### Principal findings

Total response was as high as 70 % among our underserved populations using a funnelled invitation design. Of the responders, 62 % participated in the HRA. Postal response was 41 %, of whom 66 % participated. Telephone response was 46 % among postal non-responders, of whom 59 % participated in the HRA. A face-to-face invitation barely increased response and HRA participation rates. Of the high-risk individuals, 59 % participated in the PC, irrespective of invitation step.

### Implications and future research

Underserved populations can be reached by a low-cost culturally adapted postal approach with a reminder and follow-up telephone calls. The actual cost-effectiveness of this approach needs to be studied. Efforts should be particularly targeted at GP practices in the most deprived areas, focusing on why response and participation fall behind less deprived but still low socioeconomic areas. Future qualitative (ethnographic) studies could be useful. Though a face-to-face approach barely increased response and participation, in The Netherlands, only this ‘case-finding’ approach is currently reimbursed by basic health insurance [18]. Considering the socioeconomic inequalities in health, the feasibility of implementing a culturally adapted two-step invitation strategy to increase participation in the HRA should be discussed and studied. Moreover, to increase the likelihood of cost-effectiveness of two-stage screening, as many high-risk individuals as possible need to comply with attending their GP for further testing. If feasible, the responsibility for scheduling an appointment should be shifted toward the GP practice or other healthcare organisations.

## Additional files

Additional file 1:
**Exclusion criteria.** Detailed list of exclusion criteria for study participation. (DOCX 15 kb) Additional file 2:
**(Cultural) Adaptations to invitation, HRA, and information brochure.** Detailed list of (cultural) adaptations made to invitation, HRA, and information brochure. (DOCX 1971 kb)

## Abbreviations

CMD: Cardiometabolic disease
CVD: Cardiovascular disease
DM: Diabetes mellitus
GP: General practitioner
HRA: Health risk assessment
OR: Odds ratio
PC: Prevention consultation
SES: Socioeconomic status
