# Supplementary material for: Response and participation of underserved populations after a three-step invitation strategy for a cardiometabolic health check
Source: BMC Public Health. 2015 Sep 3;15:854. doi: 10.1186/s12889-015-2139-x (PMC4558779; doi:10.1186/s12889-015-2139-x)
Supplement: Additional file 1: — Exclusion criteria. Detailed list of exclusion criteria for study participation. (DOCX 15 kb) [file 12889_2015_2139_MOESM1_ESM.docx]

**Additional file 1**

**Exclusion criteria**

- Already having one or more of the following diseases (in ICPC codes):
  - K74 ANGINA PECTORIS
  - K75 ACUTE MYOCARDIAL INFARCTION
  - K76 OTHER CHRONIC ISCHEMIC HEART DISEASES
  - K77 CONGESTIVE HEART FAILURE
  - K78 ATRIAL FIBRILLATION / -FLUTTER
  - K79 PAROXYSMAL TACHYCARDIA
  - K82 COR PULMONARY
  - K83 VALVE DISEASE NOT RHEUMATIC/NOS
  - K84 OTHER HEART DISEASES
  - K86 HYPERTENSION WITHOUT ORGAN DAMAGE.
  - K87 HYPERTENSION WITH ORGAN DAMAGE.
  - K89 TRANSIENT CEREBRAL ISCHEMIA/TIA
  - K90 CEREBROVASCULAR ACCIDENT (CVA) [EX.TIA]
  - K91 ATHEROSCLEROSIS [EX.CORON.,CEREBR.]
  - K92 OTHER DISEASES PERIFERAL ARTERIES
  - T90 DIABETES
  - T93 LIPID DISORDER
  - U88 GLOMERULONEPHRITIS/NEFROSIS
  - U99 OTHER DISEASES URINARY TRACT
- Use of one of the following drugs (in ATC-classifications):
  - A10 ANTIDIABETICS
  - B01/C01/C02/C03/C07/C08/C09 ANTIHYPERTENSIVES
  - C10 ANTILIPAEMICS
- Complete risk profile with a maximum of one year old with a known measurement for all of the following factors:
  - Smoking status
  - Comments on characteristics of diet
  - Physical activity
  - Alcohol use
  - BMI
  - Waist circumference
  - Systolic blood pressure
  - Fasting glucose
  - LDL
