# Supplementary material for: Response and participation of underserved populations after a three-step invitation strategy for a cardiometabolic health check
Source: BMC Public Health. 2015 Sep 3;15:854. doi: 10.1186/s12889-015-2139-x (PMC4558779; doi:10.1186/s12889-015-2139-x)
Supplement: Additional file 2: — (Cultural) Adaptations to invitation, HRA, and information brochure. Detailed list of (cultural) adaptations made to invitation, HRA, and information brochure. (DOCX 1971 kb) [file 12889_2015_2139_MOESM2_ESM.docx]

**Additional file 2**

**(Cultural) Adaptations to invitation, HRA, and information brochure**

Personalization

In the invitation letter, we used the patient’s demographics, such as gender, last name, and GP name. Examples:

“Dear Mr. Gül” / “Dear Ms. Gül”.

“Kind regards, your GP, H.J. van Duijn, M.D.”

Formulation

Taking into account the lower (health) literacy levels and language barriers of our population, we used short sentences and started every sentence on a new line. Example:

“Maybe you have doubts about testing your health.

“Then please read the brochure.”

Gender and ethnicity targeting

Targeting refers to designing messages for a subgroup of a population taking into account characteristics shared by the subgroup’s members, which we applied to gender and ethnicity.

- Gender- and ethnic-specific pictures (see example HRAs and brochures).
- Reference in text to specific ethnic descent. Example:

“What factors increase your risk of cardiovascular disease, diabetes, and kidney failure?

🡪 Surinamese origin.

You have an increased risk of diabetes.”

- Additional Turkish and Arabic language for Turkish and Moroccan patients (see example HRAs and brochures).
- An anecdote/story of a person with the same gender and ethnic background describing their decision-making process and what participation had brought them. A common, ethnic-specific last name was chosen for this person.
- Common barriers for these groups to screening attendance and information provision on these topics: fear for the test result; no perceived control over one’s health; no perceived effect of the test results; for Dutch and Surinamese patients experiencing no health complaints; and for Turkish and Moroccan patients already attending screening in home country.
- A sentence on the person’s right to do this check, as was found to be of importance for these groups.

| **Example native Dutch male HRA in Dutch**  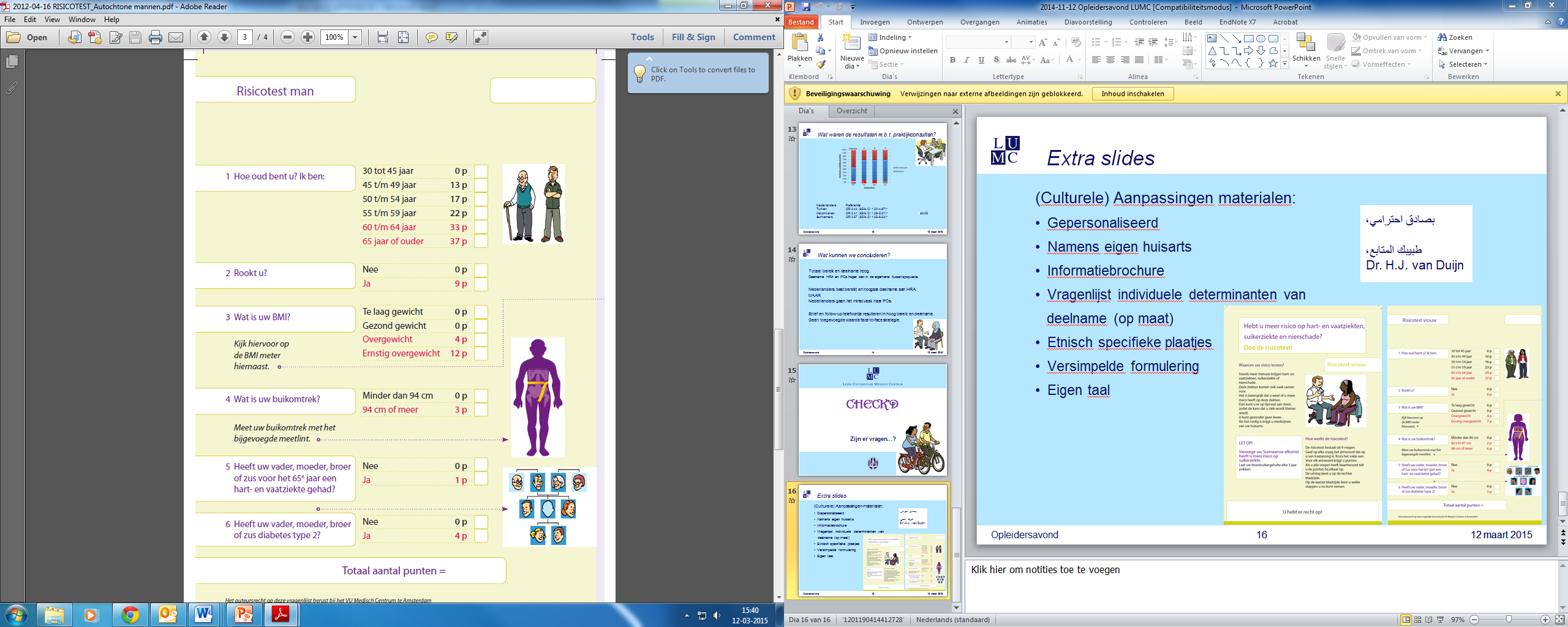 |
| --- |
|  |
| **Example Turkish female HRA in Turkish**  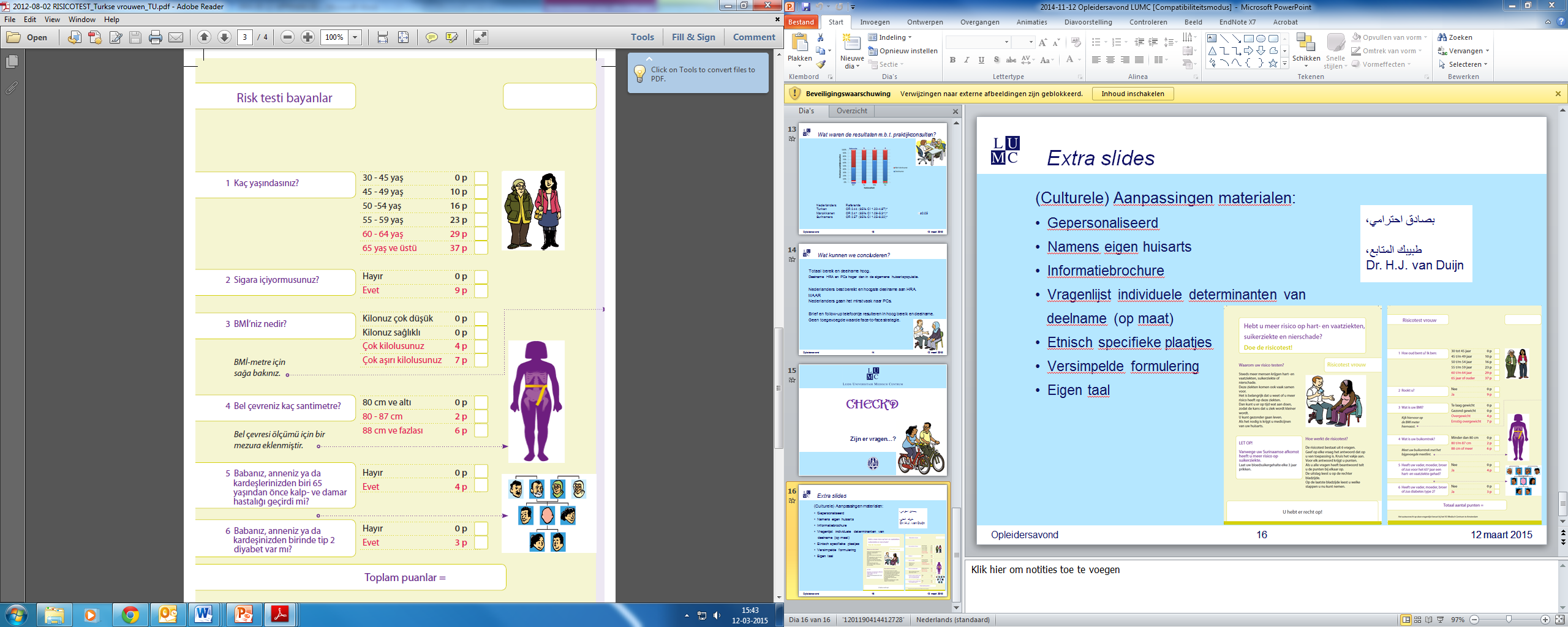 |

| **Example Surinamese female brochure in Dutch**  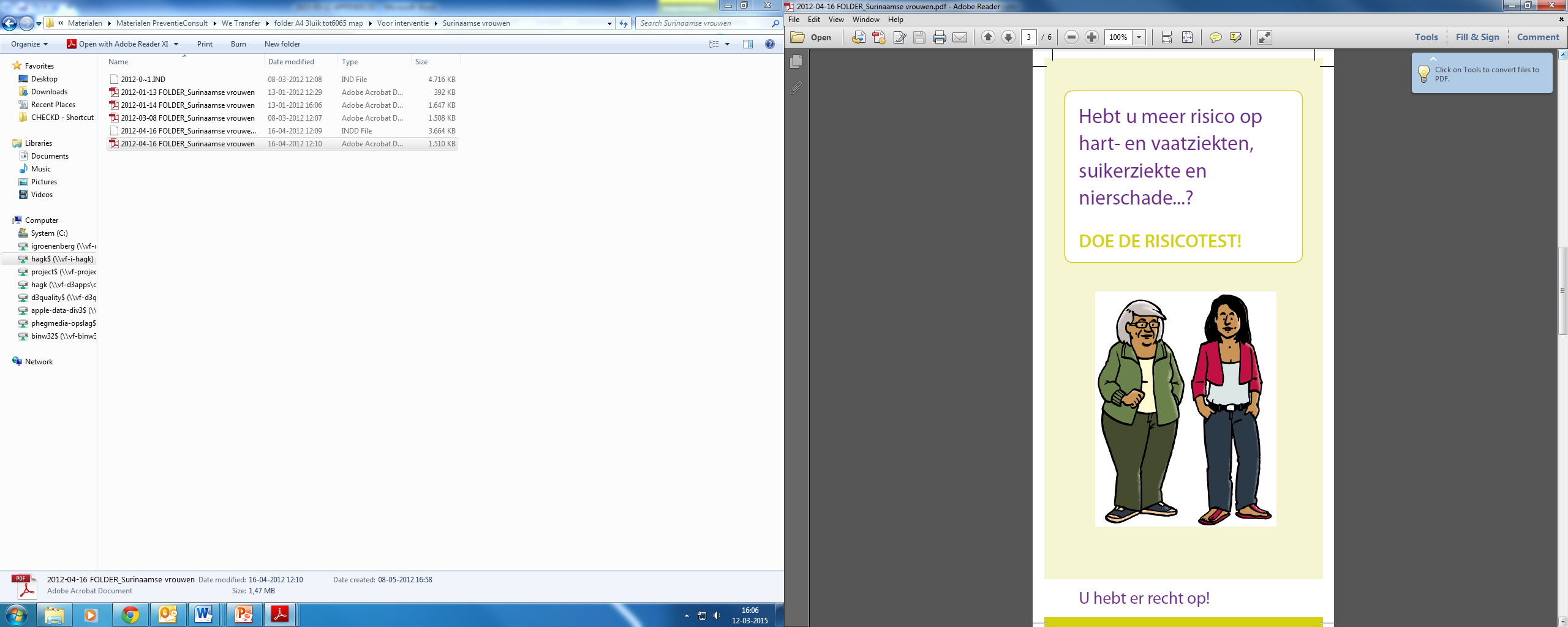 | **Example Moroccan male brochure in Arabic**  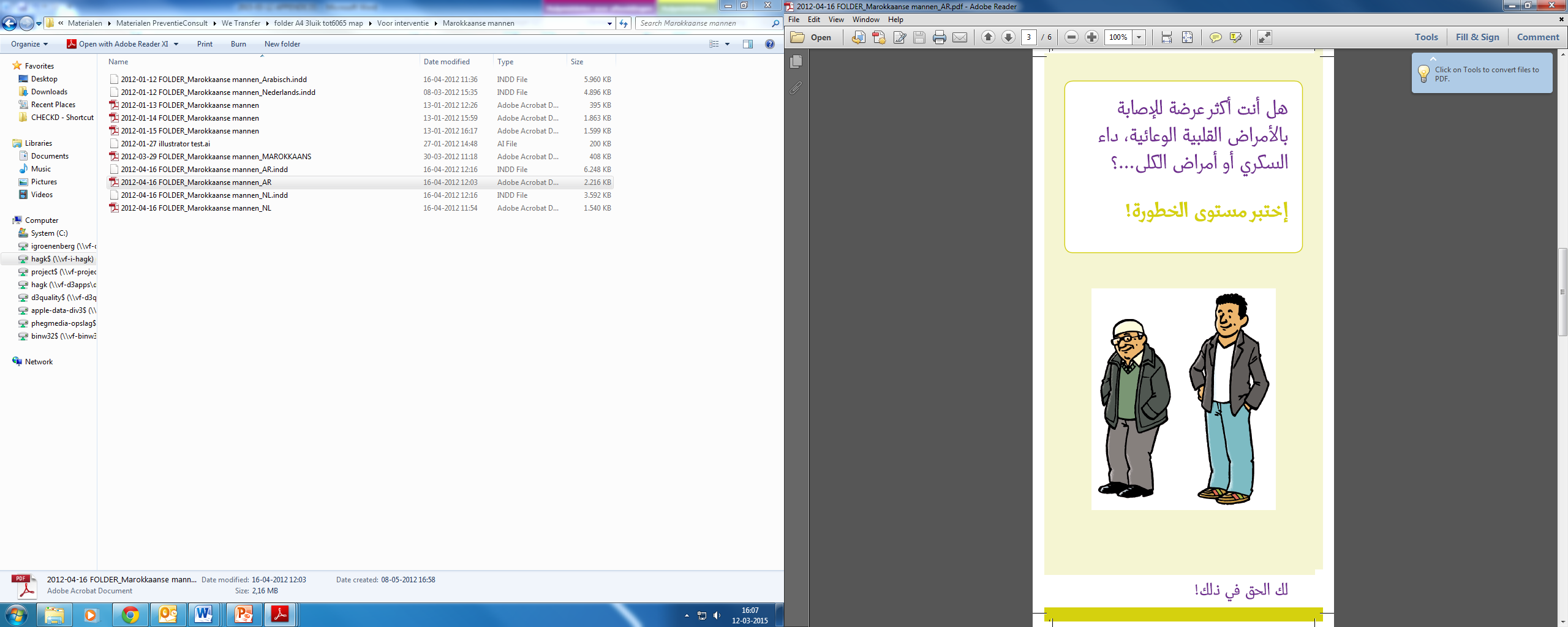 |
| --- | --- |
